# Editorial: Oxidative Stress, metabolic dysfunction and subfertility

**DOI:** 10.3389/fphys.2023.1247585

**Published:** 2023-08-29

**Authors:** Rehana Rehman, Mukhtiar Baig, Faiza Alam

**Affiliations:** ^1^ Department of Biological and Biomedical Sciences, Aga Khan University, Karachi, Pakistan; ^2^ Faculty of Medicine Rabigh, King Abdulaziz University, Rabigh, Jeddah, Saudi Arabia; ^3^ PAPRSB Institute of Health Sciences, Universiti Brunei Darussalam, Bandar Seri Begawan, Brunei

**Keywords:** oxidative stress, metabolic dysfunction, subfertility, reactive oxygen species, type 2 diabete mellitus

Frontier is a reputed platform where authors wish to contribute their latest findings and most influential original research to review articles in historically untouched areas. One such opportunity was provided by the topic “*Oxidative stress, metabolic dysfunction and subfertility,*” which published four manuscripts after a tedious peer-review process, including two review articles, one original and one systematic review.

The Research Topic aimed to cover and review the promising, recent, and novel research trends in the Research on Oxidative Stress (OS) and its effect on the body systems causing various diseases notably obesity, cardiovascular and renal dysfunctions, thyroid disorders and implications of its deficiency on reproduction. Furthermore, it encompassed the mechanisms involved in managing the oxidative environment and identifying the therapeutic agents being used.

The article by Chen et al. titled: Oxidative RNA Damage in the Pathogenesis and Treatment of Type 2 Diabetes, highlights the importance of oxidative damage to RNA leading to the pathogenesis of type 2 diabetes mellitus (T2DM) and its impediments.

The paper discusses the magnitudes and processes of intracellular response to RNA oxidation, reviews the association of reactive oxygen species (ROS) with T2DM, and use of antioxidants as one of the approaches for the management of T2DM, although the pharmacological effects remain unclear.

The limitations of this paper are not explicitly stated. Additionally, the paper does not provide a comprehensive overview of all the factors contributing to the development of T2DM, and further research is needed to fully understand the disease’s complexity.

The paper suggests that future research should focus on developing novel complexes that inhibit reactive oxygen-producing enzymes, increase intracellular antioxidant defense, and control the production of free radicals. Additionally, the causality of oxidative RNA damage in T2D patients and fundamental regulatory mechanisms of glucose metabolism and insulin sensitivity needs to be explored. The paper furthermore suggests that more clinical trials are needed to evaluate the pharmacological effects of antioxidants in managing T2DM.

Zhu et al.’s article titled “Effects of acupuncture on the pregnancy outcomes of frozen-thawed embryo transfer: A systematic review and meta-analysis” investigated outcomes of pregnancy after acupuncture given to females who undergo Frozen Embryo Transfer (FET). It states that clinicians and patients can consider acupuncture a safe therapy to increase the chances of conception after FET. However, the value of evidence remains inadequate due to the limited sample size and quality of trials. Therefore, future RCT on a large scale, investigation of acupuncture protocols and comparison of fresh with FET cycles is recommended.

The impact of infertility stress on sexual function in infertile men and women was emphasized by Amraei et al. in their study “Does Infertility Stress Impair Sexual Function in Infertile Women and Men? A Cross-Sectional Study in Iran”. The study describes that the stress of infertility was more evident in infertile females than infertile male subjects. The infertile females experienced low sexual self-esteem and sexual relationship with the increase in the duration of infertility and the financial burden of paying for infertility treatment costs. On the contrary, infertile men had more Research Topic in communication. The article suggests that policymakers should consider counselling strategies for the couple to enable them to cope with their psychological Research Topic. However, the study has limitations like lack of enrollment of couples, the nonexistence of a control group and the element of recall bias since the information was collected on a questionnaire.

Pan et al.’s review paper provides an inclusive summary of the regulatory patterns of Sirtuins at multiple levels and their role in the regulation of inflammation, OS, bone metabolism and microbial activity. The authors summarize the regulatory mechanisms of Sirtuins and suggest their potential as curative targets in oral diseases, particularly periodontitis and oral cancer. The authors suggest that the information presented in the paper could lead to developing new interventions for oral diseases based on targeting Sirtuins. Further research is required to explore the functions of other Sirtuins members in oral diseases and to address several Research Topic in this field regarding the roles of Sirtuins in oral squamous cell carcinoma.

Recent years have seen growing signs of the impact of OS and metabolic dysfunction on both genders’ fertility (Hussain et al., 2023; Raut and Khullar, 2023). The literature shows the outcome of these elements on infertility yet requires further evidence. It is currently suggested that ROS damages sperm cells and oocytes, creating greater complexity with fertilization besides affecting ovaries and testis functionality, leading towards the compromised output of healthy eggs and sperm. A healthier lifestyle can aid in alleviating OS, including balanced nutrition, regular exercise, deterring tobacco smoking and reducing alcohol consumption, and supporting optimal metabolic health to enhance fertility (Carnevale et al. 2018; Sharma and Mehdi, 2023). Moreover, certain studies suggest antioxidants may help improve reproduction outcomes by reducing OS effects.
